# “It’s No Choking Matter!”: The Search for Evidence Relating to Home-Delivered, Texture-Modified Meals – A Systematic Literature Review

**DOI:** 10.1007/s13668-026-00755-3

**Published:** 2026-04-23

**Authors:** Alison Host, Karen Charlton, Karen Walton

**Affiliations:** 1 Faculty of Science, Medicine and Health, University of Wollongong, Wollongong, NSW Australia; 2 School of Health Sciences, University of Newcastle, Callaghan, NSW Australia; 3 Nutrition and Metabolic Health, Hunter Medical Research Institute, New Lambton Heights, NSW Australia

**Keywords:** Home-delivered meals, Texture-modified meals, Dysphagia, Community, Older adults, Ageing in place

## Abstract

**Purpose of Review:**

Home-delivered meals have been shown to confer benefits conducive to successfully ageing in place. However, little is known about home-delivered, texture-modified meals designed for older community-dwelling people with dysphagia, despite recognition that such individuals are at significant nutritional risk. The purpose of this review is therefore to systematically identify, appraise, and synthesise any existing evidence relating to home-delivered, texture-modified meals for community-dwelling persons with dysphagia.

**Recent Findings:**

A systematic search of the literature was performed across five relevant databases (CINAHL, Medline, Pub Med, Scopus, and Web of Science) to answer the research question, “What is currently known about home-delivered, texture-modified meals for community-dwelling individuals with dysphagia?”. Broad inclusion criteria and minimal limits were applied. Articles were included if they reported upon any outcomes specific to home-delivered, texture-modified meals. Following removal of duplicates, 521 articles were subjected to screening by title and abstract, and 40 to full-text review. No studies of specific relevance to the research question were returned.

**Summary:**

The findings of this systematic literature review indicate a true gap in the literature and a need to establish a baseline of evidence about home-delivered, texture-modified meals. Doing so will help to ensure that community-dwelling older persons with dysphagia have equitable access to safe and nutritionally adequate meals that support successful ageing in place.

**Supplementary Information:**

The online version contains supplementary material available at 10.1007/s13668-026-00755-3.

## Introduction

Global population-ageing and the related increase in age-related chronic illness and disability, including dementia, have prompted an escalation in demand for both health and aged-care services that presents a major challenge for governments and policymakers [1–3]. In response, strategies designed to support older adults to “age in place” (i.e. allow them to remain living safely and independently within their own home for as long as possible) [3, 4], are being implemented to thwart, or at least delay, the need for permanent placement in residential care, thereby reducing healthcare costs [1, 2]. “Ageing in place” promotes better health and independence in a manner that confers personal choice, respect and dignity, and that helps to maintain social connectedness [1, 2, 4]. This approach makes good sense, both from an economic standpoint and from the perspective of older persons themselves, who have broadly expressed their preference to remain living independently in the comfort of their own home [1, 2, 5].

Older populations are vastly heterogenous, varying widely in terms of their individual needs for care and support [3, 6]. In Australia, lower-level assistance with activities of daily living may be accessed through the entry level Commonwealth Home Support Program [7–10]. However, older people who require a higher-level of care and support to remain living at home are referred for assessment under the more comprehensive and structured Home Care Packages Program, which comprises four levels of support ranging from basic to high care needs [7–10]. In line with recommendations from a recent Royal Commission into Aged Care Quality and Safety (2018–2021), a new Aged Care Act (2024) will be implemented in a staged manner from November 2025 that will result in the amalgamation of these two programs into a single new Support at Home Program [11]. Under this new program, participants will be assessed both for eligibility and the level of support required, and subsequently allocated a budget to partially or fully subsidise in-home support services [1, 11, 12]. Other countries that have adopted similar government-subsidised, means-tested user-contribution aged care models include Canada, New Zealand, Singapore, Japan, England, France, Norway, Finland, and Spain; however, individual models and levels of government spending on aged care (as a percentage of gross domestic product [GDP]) vary between countries [13–17].

One service that may be accessed under the two existing support programs is home-delivered meals. This is a popular choice amongst older Australians receiving in-home support [8, 18]. In the year 2022-23, 12.6% (*n* = 102,426) of all Home Support recipients subscribed to meal services, rendering this the seventh most commonly received form of support [8]. Ample evidence from multiple countries substantiates the multiple benefits provided by good quality food and nutrition in the later years of life. These include the maintenance of health and independence, a lower risk of malnutrition and its related complications such as falls and hospitalisation, support of cognitive function and positive mood, and opportunities to remain socially connected [2, 19]. Similarly, research conducted with recipients of home-delivered meals has demonstrated improved nutritional intake and/or reduced risk for malnutrition [2, 4, 19–28], reduced food insecurity [2, 19, 24, 25, 29, 30], fewer falls [2, 25] and lower rates of hospitalisation [2, 31], greater maintenance of independence [2, 4, 19, 25, 29, 32], and reduced loneliness [4, 19, 25, 29, 33]. There is also some evidence that home-delivered meals can be appropriately tailored for those with chronic health conditions, such as heart disease and hypertension, cancer, diabetes, chronic obstructive pulmonary disease and chronic kidney [34–36]. However, it remains unclear how well home-delivered meal services cater for people with identified swallowing difficulties who require texture-modified options.

Dysphagia, or swallowing dysfunction, is a problem commonly faced by older adults [37–42], with as many as 30% of community-dwelling people aged over 65 years, and more than half of those in residential care, thought to be affected [42–48]. Dysphagia may arise as a consequence of various chronic diseases or the ageing process itself [38, 40, 47, 49, 50], although is particularly common amongst those with dementia and frail older adults [40, 42, 45, 49, 50]. People with dysphagia experience difficulty with eating and drinking which, if not managed, incurs significant risk for choking and asphyxiation, aspiration, pneumonia, and in the most serious of cases, respiratory failure or death [38, 42, 43, 51]. Longer term, there exists a greater likelihood for weight loss, dehydration, malnutrition and associated poor outcomes for health, including anaemia, infection, falls, the development of pressure injuries, and a more frequent need for medical treatment and longer hospital admissions [38, 40, 42, 50, 52, 53]. Community-dwelling older persons with self-reported dysphagia are also more likely than those who do not report swallowing problems to be homebound or semi-homebound and food insecure [54]. Hence, for these older people, home-delivered meals may provide an important means for accessing adequate nutrition.

Treatment for dysphagia may involve a range of strategies to compensate for identified swallowing limitations, but will often include individualised recommendations for dietary texture modification [38, 42, 49, 55–60]. Modification typically involves thickening of fluids, and/or reduction of solid food textures by chopping, mincing, mashing or pureeing [43, 58, 60]. However, the term may also be applied to the exclusion of particular textures or food items which prove to be problematic [25]. Texture-modified foods and fluids are commonly categorised according to the evidence-based International Dysphagia Diet Standardisation Initiative (IDDSI) standards for foods and fluids, which were developed in 2015 to provide an internationally uniform language for describing the different food and fluid textures and consistencies [55, 59]. This system has since been broadly accepted around the globe and replaces former country-specific terminologies [61].

While adherence to a texture-modified diet may mitigate the potential for adverse outcomes, several limitations are apparent. Of significant concern is the propensity for foods and fluids of a modified consistency to be poorly tolerated by recipients due to factors such as reduced sensory appeal and mouthfeel, altered taste, variation in the resultant consistency, and embarrassment [42, 51, 56, 62]. This may lead to an overall reduction in oral intake (and hence greater nutritional risk) and an increased likelihood for textural non-compliance [42, 52, 56]. Evidence also shows that meals with modified consistencies are typically marked by limited choice and variety, lower energy and protein content and intakes, and greater nutritional inconsistency upon reproduction than their regular-textured counterparts [42, 52, 56, 62–65]. These findings are of clinical significance, since individuals with dysphagia often present with multiple comorbidities and a higher incidence of cognitive decline and self-feeding limitations [43, 47, 49, 50, 63, 66, 67], that in themselves may serve to increase nutritional requirements, negatively impact oral intake and predispose risk for malnutrition. Risk is further intensified in older persons who may be experiencing multi-factorial age-related physical, pathological, psychosocial and environmental changes that compromise dietary intake and increase needs for protein and other nutrients, including iron, zinc, calcium and vitamins B6 and D [28, 42]. Cumulatively, such health and lifestyle factors may incite social withdrawal, depression and an overall reduction in quality of life [40, 42, 68, 69], thereby exacerbating existing risk to health and wellbeing.

Demographic ageing of populations worldwide, coupled with increases in both the incidence of dementia and a growing demand for in-home aged care support services, indicates an escalating number of older persons likely to require in-home support to manage dysphagia, for whom one of the most important supports will be home-delivered, texture-modified meals. Therefore, the aim of this paper is to systematically identify, appraise and synthesise the current body of evidence regarding home-delivered, texture-modified meals for community-dwelling people with dysphagia.

## Methodology

This Systematic Literature Review (SLR) was registered with the National Institute for Health and Care Research (NIHR) online international systematic review registry (Prospero) on 3rd April 2025 (ID 1025148), and findings reported in accordance with the Preferred Reporting Items for Systematic Reviews and Meta-Analyses (PRISMA) Guidelines 2020 [70]. Searches were repeated in five databases (Medline, CINAHL, Scopus, Pub Med and Web of Science), with the most recent search conducted on 7th April, 2025.

This review sought to address the following research question: What is currently known about home-delivered, texture-modified meals for community-dwelling individuals with dysphagia? The research question and subsequent search terms were deliberately designed to be as broad as possible, in order to capture any research about home-delivered, texture-modified meals that had been published within the previous ten years. This was to ensure recency of data and inclusion of the time period from just prior to the introduction of the IDDSI guidelines [71] until the present time.

A university librarian was consulted, and the search strategy refined so as to maximise the number of articles returned. Due to the broad nature of texture-modification, the focus for the search was contained to foods, and did not include fluids. Search terms were devised to capture the differing language typically used on an international basis to describe the various modified meal textures or related diet descriptors (e.g. “dysphagic” diet), both prior to and following the implementation of the IDDSI standards. Articles were otherwise limited only to full text articles and those published in English. No limits were applied to the gender or medical status of participants, country of research, study design, or source of publication. However, additional limits to the subject area and several keywords of relevance to the topic were introduced for the Scopus database due to an otherwise unmanageable number of irrelevant articles being returned. Articles were deemed eligible if they reported any outcome specific to home-delivered texture-modified meals, including (but not limited to) availability, accessibility, menu variety, textures offered, cost implications, nutritional value, consumer satisfaction and quality of life. Articles were excluded only if they failed to specifically address any aspect of home-delivered meals of a modified texture, whether they reported only upon meals of a regular texture, or were conducted within clinical, rehabilitation or long term care settings. Full details of the search strategy are provided in Supplementary Table 1.

Articles returned by the search strategy were imported into Covidence for processing, and duplicates automatically removed. Remaining articles were screened by title and abstract (AH), and potentially eligible articles subjected to a full-text review in duplicate (AH and KW). Discrepancies were referred to a third reviewer (KC) for resolution.

## Results

**Fig. 1:**
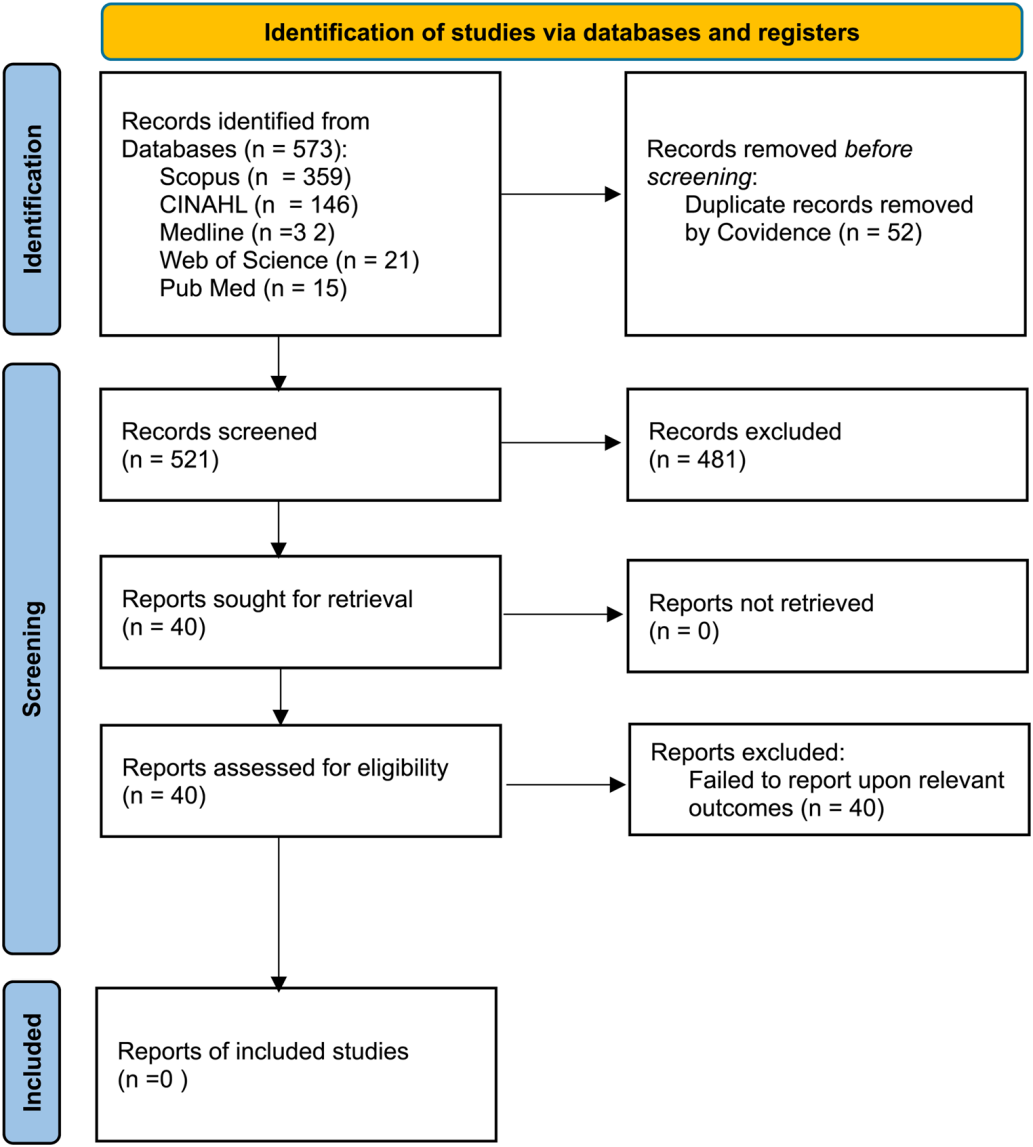
PRISMA flow diagram. Source: Page MJ, et al. .The PRISMA 2020 statement: An updated guideline for reporting systematic reviews. BMJ 2021; 372:n71. doi: 10.1136/bmj.n71. Licenced under the Creative Commons license (CC BY 4.0)

Application of the search strategy to all five of the selected databases generated a total of 573 articles (Scopus 359, CINAHL 146, Medline 32, Web of Science 21 and Pub Med 15). Following the removal of duplicates, 521 articles remained for screening by title and abstract, of which 40 were subjected to a full text review. Despite the application of extremely broad inclusion criteria, no articles were found to be eligible for quality and content analysis due to a lack of reported outcomes specifically related to home-delivered, texture-modified meals, as shown in the PRISMA flow chart (Fig. 1).

## Discussion

The intention of this systematic literature review was to identify, appraise, and synthesise any existing empirical evidence relating to home-delivered, texture-modified meals for community-dwelling older adults with dysphagia. Despite this systematic approach, no articles of specific relevance to the research question were found. This review has therefore identified a true gap in the published literature with respect to home-delivered meals.

Screening of the literature indicated that much of the published literature concerning texture-modified foods has addressed technical attributes and processing techniques, or factors with the potential to influence either nutritional intake or appetite. Examples include the use of food moulds, 3D and 4D printing of foods, molecular gastronomy, chemosensory stimulation, fortification, the prescription of oral nutritional supplements, and the application of non-thermal technologies and artificial intelligence (AI) mathematical modelling [46, 51, 72–77]. More recently, there has also been some information published about the effectiveness of texture-modified foods and thickened fluids for managing risk, and the associated impact of these products upon the client’s quality of life [78, 79].

However, while certainly meritorious, these studies were primarily conducted in a laboratory, acute care (hospital) or long term care (residential aged care or nursing home) setting. This is despite acknowledgement in the literature that a significant proportion of home-delivered meal recipients (and, hence, community-dwelling individuals) report difficulty with chewing and swallowing or dental issues (for example, [27, 80, 81]). Similarly, published research related to home-delivered meals and home-delivered meal programs has occurred largely to the exclusion of people with chewing or swallowing difficulties (e.g. [82–85]) and those who require texture-modified meals or a restrictive diet (e.g. [86–88]), or without assessing and reporting the swallowing capacity of participants (e.g. [21, 24, 29, 30, 34, 35, 89–92]). In cases where people with swallowing difficulties or dysphagia were included, this was self-reported (e.g. [26, 54, 80, 81]) rather than objectively assessed. In those studies, outcomes specific to these participants and details about the nature of meals consumed were not reported (e.g. [26, 36, 80, 81, 90, 93]). This seems counterintuitive, given the widely reported high risk health status of people with dysphagia [40, 42, 43, 47, 49–54, 65–67] and multiple problems with texture-modified diets observed within both acute and long term care settings [42, 50, 52, 56, 62, 64, 94].

While there are some guidelines that address food and nutrition in older age, and which include recommendations regarding dysphagia, home-delivered meals and texture-modified foods (for example, [95, 96]), research into the application these guidelines for older people who meet all three conditions is absent.

Dysphagia is often associated with the existence of comorbidities and frailness, and as a result, a high risk of malnutrition and its related complications [38, 42, 49, 52, 66, 97]. Catering for a texture-modified diet also carries a substantial burden, particularly when there is a requirement for the more highly modified textures. Not only is there a need for extra time and care during food preparation, but also the added responsibility of ensuring that the resultant meal is appetising, nourishing and compliant with the prescribed textural requirements so that it will not pose a risk to the client’s safety. For people who are already unwell, preparing their own texture-modified meals may not be a plausible option, and having access to pre-prepared texture-modified meals may become a requirement. Even for individuals who have access to personal support networks, the responsibility and additional burden bestowed upon carers is significant. This point has been recognised and acknowledged in the literature [3, 4, 68, 71, 97–99], and should not be overlooked.

In view of the sustained trend towards population ageing and greater incidence of dementia [2, 6], an increase in the number of older people requiring dysphagia management may be anticipated. With the current move towards ageing in place, demand for home support is also likely to continue to grow [2, 100]. This is especially true for frail older adults (including those with dysphagia and cognitive deficits), as well as those discharged from hospital to home, with limited mobility and dexterity, or who lack a carer or familial and local community support. For these individuals, home-delivered meal services may be the only way they can access quality, nutritious meals [2, 29, 97] that support some - or all - of their nutrition needs.

In line with the principles of the UN Decade of Healthy Ageing Plan for Action [101], people with dysphagia should have the same rights as those without swallowing limitations to access high quality services that support their ability to age in place, including home-delivered meals. Likewise, carers of individuals with dysphagia should have the right to access services that will provide respite and help alleviate concerns [3] about ensuring safe, nutritious and appetising meals within the home. Research to support such initiatives, primarily for the benefit of the consumers, is long overdue.

To be clear, the fact that this review did not uncover any published studies relating to home-delivered, texture-modified meals does not equate to there being a lack of available services, nor cast any aspersions regarding the quality of such services if they do exist. The availability and quality of texture-modified, home-delivered meal services represent a critical yet underexamined component of supportive care for this population. Moreover, such services may constitute an emerging area of commercial and public health significance. Empirical research to inform the development, implementation and evaluation of these services, principally to support adequate consumer intake, is required.

This review therefore raises critical questions regarding the availability and adequacy of services for individuals requiring texture-modified diets. It remains unclear whether existing home-delivered meal programs adequately cater to this population or whether such meals meet appropriate standards for nutritionally quality, safety, palatability and affordability. Addressing this gap requires establishing a baseline of evidence to guide future research and identify areas necessitating further investigation of policy action.

We propose the development of robust research protocols that enable the inclusion of community-dwelling older adults requiring home-delivered, texture-modified meals, with outcomes specific to this cohort. The presence of dysphagia should be consistently assessed, reported, and where possible, confirmed by a Speech Pathologist (internationally referred to as, for example, Speech-Language Pathologist or Therapist, Speech and Language Therapist, Logopaedist, or Phono-audiologist) using standardised methods.

A key strength of this review lies in its comprehensive search strategy, encompassing five databases and inclusive of international terminology reflecting both pre-IDDSI and IDDSI frameworks. Minimal restrictions were applied to maximise the identification of relevant studies across countries, study designs and participant characteristics. Limitations include the restriction to English language publications and exclusion of research published prior to 2015. However, earlier literature would not have reflected current practice or international IDDSI standards.

The decision to conduct a systematic literature review (SLR) was deliberate, guided by the objective of identifying empirical evidence relating to home-delivered, texture-modified meals for community-dwelling individuals with dysphagia, rather than mapping broader concepts or service models. A systematic approach was therefore considered the most rigorous way to determine whether such evidence exists, and confidently establish its absence if no evidence could be found. Consequently, the lack of eligible studies returned by this review reflects a genuine evidence gap, as opposed to a limitation of the methodology, and highlights an important priority for future research. Supplementary scoping of the grey literature may provide a useful extension to this review.

## Conclusion

With global population ageing, increasing dementia prevalence and policy shifts towards ageing in place, the demand for home-delivered, texture-modified meal services is expected to rise. This review identifies a substantial evidence gap concerning the availability, accessibility, nature and adequacy of such services and underscores an urgent need to establish a foundational evidence base to inform future research and practice.

## Key References

- Fleury S, Tronchon P, Rota J, Meunier C, Mardiros O, Van Wymelbeke-Delannoy V and Sulmont-Rossé C. The nutritional issue of older people receiving home-delivered meals: A systematic review. Front. Nutr. 2021a; 8:629580. doi:10.3389/fnut.2021.629580
  - ○This recent systematic literature review summarised the evidence with respect to nutritional status and nutritional intake of home-delivered meals recipients. Results underscored the high nutritional risk and inadequate nutritional intakes experienced by home-delivered meal recipients, as well as the beneficial impact of home-delivered meals on nutritional status and nutrient intake.
- Walton K, do Rosario VA, Pettingill H, Cassimatis E, Charlton K. The impact of home-delivered meal services on the nutritional intake of community living older adults: a systematic literature review. J Hum Nutr Diet. 2019; 33(1):38–47. doi:10.1111/jhn.12690
  - ○This systematic literature review examined the evidence with respect to the impact of home-delivered meals on the dietary intake of older community-dwelling people on days where meals were received compared to days when meals were not received. Results indicated that home-delivered meals improve recipients’’ dietary intake for energy, protein and micronutrients, including vitamins A, B complex and D, and the minerals calcium, zinc, and magnesium.
- Wu XS, Miles A, Braakhuis A. Nutritional intake and meal composition of patients consuming texture modified diets and thickened fluids: A systematic review and meta-analysis. Healthcare. 2020; 8(4):579. doi: 10.3390/healthcare8040579
  - ○This systematic literature review and meta-analysis summarised the evidence with respect to both the limitations inherent to texture-modified diets and thickened fluids, and also nutrition outcomes for adults consuming texture-modified diets and thickened fluids. All 35 studies included were conducted in hospitals, long-term care facilities or a combination of both. Results highlighted the nutritional inadequacies and discrepancies between regular and texture-modified diets, problems associated with texture, impact of texture-modified diets and thickened fluids on oral intake, and effects of using food-shaping and fortification techniques, and oral nutrition supplements (ONS). The challenge of differing terminologies prior to the implementation of the International Dysphagia Diet Standardisation Initiative (IDDSI), as well as the inclusion of mixed populations with varied medical conditions, was also discussed.

## Supplementary Information

Below is the link to the electronic supplementary material.

Supplementary Material 1

## Data Availability

No datasets were generated or analysed during the current study.
