# Supplementary material for: “It’s No Choking Matter!”: The Search for Evidence Relating to Home-Delivered, Texture-Modified Meals – A Systematic Literature Review
Source: Curr Nutr Rep. 2026 Apr 23;15(1):38. doi: 10.1007/s13668-026-00755-3 (PMC13106258; doi:10.1007/s13668-026-00755-3)
Supplement: Supplementary file 1 — Supplementary Material 1 [file 13668_2026_755_MOESM1_ESM.docx]

| **Criteria** | **Details** |
| --- | --- |
| Databases | - CINAHL (Search field “optional) - Medline Full Text (Search field “optional”) - Pub Med (“All Fields”) - Scopus (“all fields”) - Web of Science (“all fields”) |
| Search Terms | Line 1: “home delivered” OR “home delivery” OR “Meals on Wheels”  Line 2: “texture modified” OR puree* OR mince* OR mash* OR blend* OR vitamiz* OR vitamis* OR ground OR homogeniz* OR homogenis* OR smooth OR soft* OR dysphagi* OR deglutition OR swallow*  Line 3: meal* OR food*  Line 4: S1 and S3  Line 5: S2 and S4 |
| Limits applied | - Full Text (except Web of Science, as function not available) - Language: English - Year of publication: 2015 to 2025   For Scopus only,   - Subject Area: medicine, social sciences, nursing, multidisciplinary and health professionals - Keywords: human, humans, adult, aged, home delivery, malnutrition, diet, catering service, nutrition, nutritional status, very elderly, quality of life, food security, food supply, caloric intake, aged 80 and over, nutritional assessment, dietary intake, public health, food security, older adults, food intake, food, dysphagia, meals, health care delivery, feeding behavior, diet therapy, nutrition assessment, eating, food services, food assistance, energy intake, aging, health care access, social support, home delivered meal, health behavior, nutritional value, health service |
| Inclusion Criteria | - Articles published in English. - Articles published between 2015 to 2025 (inclusively). - All study types. - Studies reporting upon any outcomes related to home-delivered meals for dysphagia or with modified meal textures (soft, minced, pureed or equivalent thereof). - Participants from any age, gender or country demographic. - Participants with or without additional health problems or medical diagnoses. |
| Exclusion criteria | - Articles not published in English. - Articles published prior to 2015. - Studies which did not report outcomes specific to texture-modified, home-delivered meals. - Reports of impending studies, yet to be undertaken. |

**Supplementary Table1:** Search strategy.
